# Hepatic perfusion as a new predictor of prognosis and mortality in critical care patients with acute-on-chronic liver failure

**DOI:** 10.3389/fmed.2022.1008450

**Published:** 2022-10-10

**Authors:** Johannes Vogg, Constantin Maier-Stocker, Stefan Munker, Alexander Mehrl, Sophie Schlosser, Hauke Christian Tews, Karsten Gülow, Martina Müller, Stephan Schmid

**Affiliations:** ^1^Department of Internal Medicine I, Gastroenterology, Hepatology, Endocrinology, Rheumatology and Infectious Diseases, University Hospital Regensburg, Regensburg, Germany; ^2^Department of Anesthesiology and Critical Care, School of Medicine, Technical University of Munich, Munich, Germany; ^3^Department of Medicine II, University Hospital, LMU Munich, Munich, Germany

**Keywords:** liver disease, liver cirrhosis, acute-on-chronic liver failure, liver perfusion, doppler ultrasound, critical care medicine

## Abstract

**Background and aims:**

Liver diseases are frequent causes of morbidity and mortality worldwide. Liver diseases can lead to cirrhosis, with the risk of acute-on-chronic liver failure (ACLF). For the detection of changes in hepatic hemodynamics, Doppler ultrasonography is a well-established method. We investigated hepatic hemodynamics *via* serial Doppler ultrasonography to determine the predictive value of changes in hepatic perfusion for the outcome in patients with severe liver diseases compared to established prognostic models such as the MELD (Model for End-Stage Liver Disease) or CLIF-C (Chronic Liver Failure-Consortium) ACLF score.

**Methods:**

In this prospective cohort study, hepatic perfusion was quantified at baseline before the initiation of treatment and every third day by means of serial measurements of the hepatic artery resistance index (HARI) and the maximum portal vein velocity (PVv) using Doppler ultrasonography in 50 consecutive patients with severe liver diseases admitted to a medical intensive care unit (MICU). The recorded hemodynamic parameters were compared to the MELD score, and the CLIF-C ACLF score to analyze their utility for the prediction of the outcome of patients with severe liver diseases, liver cirrhosis, and ACLF.

**Results:**

The changes (delta) obtained by serial measurements of the MELD score, HARI, and PVv were analyzed through scatter plots. Bivariate correlation analysis yielded a new positive linear correlation between the delta-HARI and the delta-MELD score (*r* = 0.469; *p* < 0.001). In addition, our data revealed a new negative linear correlation between delta-PVv and the delta-MELD score (*r* = −0.279, *p* = 0.001). The leading cause of MICU mortality was acute-on-chronic liver failure (ACLF). A subgroup analysis of patients with liver cirrhosis revealed a positive linear correlation between the delta-HARI and the delta-CLIF-C-ACLF score (*r* = 0.252, *p* = 0.005). Of clinical relevance, non-survivors of ACLF exhibited a significantly higher mean value for the delta-HARI (0.010 vs. −0.005; *p* = 0.015) and a lower mean value for the delta-PVv (−0.7 vs. 1.9 cm/s; *p* = 0.037) in comparison to survivors of ACLF.

**Conclusion:**

This study shows the prognostic value of the assessment of hepatic perfusion in critical care patients with severe liver diseases by bedside Doppler ultrasound examination and its utility as an accurate predictor of the outcome in patients with ACLF. Increasing HARI and a decreasing PVv are predictors of an adverse outcome. Delta-HARI and delta-PVv are new biomarkers of prognosis and ACLF-related mortality in patients with liver diseases. Delta-HARI and delta-PVv may be helpful in guiding clinical decision-making, especially in catecholamine and fluid management.

## Introduction

Liver diseases are significant causes of morbidity and mortality worldwide (1). The progression of liver diseases to cirrhosis and decompensation associated with critical illness is a significant cause of mortality in these patients (2, 3). Acute-on-chronic liver failure (ACLF) can occur in patients with liver cirrhosis and is a recently described entity diagnosed in patients with chronic liver diseases and a combination of hepatic and extrahepatic organ failures (kidney, respiratory, coagulation, circulatory, brain). Early diagnosis and treatment of ACLF are essential for the outcome of these critically ill patients (4, 5).

Organ perfusion plays an essential role in liver diseases, while the mechanisms regulating hepatic perfusion in patients with liver diseases, fibrosis, cirrhosis, and ACLF are only partially known (6). Portal venous flow depends mainly on the influx of splanchnic perfusion (7).

In contrast to portal venous flow, arterial flow is subject to pressure-dependent autoregulation. A second mechanism termed hepatic artery buffer response (HABR) is a central mechanism in the intrinsic regulation of hepatic blood flow (8). HABR describes the ability of the hepatic artery to compensate for changes in the flow of the portal vein by counter-directed hemodynamic adjustment of its perfusion. HABR causes arterial vasodilation through reduced portal flow and, vice versa, arterial vasoconstriction through increased portal flow (9). HABR is also preserved in patients with inflammatory liver diseases, even in advanced liver fibrosis and cirrhosis (10, 11).

Abdominal and Doppler ultrasonography are well-established methods for evaluating liver diseases and detecting changes in hepatic hemodynamics. International guidelines recommend using ultrasound scans to evaluate liver diseases (2, 3). Ultrasound scans are an easily accessible, inexpensive, and non-invasive procedure, which can be repeated as often as needed, even at the bedside of critical care patients. Such scans have high specificity in diagnosing liver fibrosis, liver cirrhosis, and portal hypertension. The diagnosis of liver cirrhosis by conventional ultrasound is based on changes in liver morphology and signs of portal hypertension. In addition, Doppler ultrasonography is a valuable tool for evaluating hemodynamic changes in cirrhotic liver tissue (12).

Little is known about the predictive value of serial measurements of hepatic hemodynamics in patients with severe liver diseases, particularly in patients with acutely decompensated cirrhosis at risk of developing acute-on-chronic liver failure.

The aim of this study was 1. to determine the predictive value of changes in hepatic perfusion for the outcome in patients with severe liver diseases compared to well-established prognostic models such as the MELD or CLIF-C ACLF score in the context of critical care treatment (13, 14), 2. to analyze the role of liver perfusion as an early predictor of mortality due to ACLF, and 3. to identify potential new hemodynamic targets in critical care for early therapeutic intervention in ACLF.

## Materials and methods

### Study design and patient characteristics

This prospective cohort study enrolled 50 patients with severe acute and chronic liver injury (ALI and CLI) and acute-on-chronic liver failure (ACLF) (Table 1). The diagnosis of liver cirrhosis was based on non-invasive tests following the current European Association for the Study of the Liver (EASL) Practice Guidelines on non-invasive tests for evaluation of liver disease severity and prognosis (15). Accordingly, we diagnosed liver cirrhosis by detecting specific morphological changes of the liver by ultrasound and computed tomography (CT) in combination with examination of clinical and laboratory chemistry parameters (16, 17). In our cohort, liver cirrhosis was diagnosed in 36 of the 50 patients studied. The patients were treated in a medical intensive care unit (MICU) of a German University Hospital that specializes in the treatment of liver diseases.

**Table 1:** Clinical characteristics of the study cohort.

**Characteristics**	**Total study cohort (*n* = 50)**
Age [years]: mean ± SD (range)	59.7 ± 10.3 (39–90)
**Sex:** ***n*** **(%)**	
Female	16 (32)
Male	34 (68)
MICU stay [days]: mean ± SD (range)	15.3 ± 13.8 (2–72)
**Mortality in the MICU:** ***n*** **(%)**	
Deceased patients^a^	16 (32)
Survived patients	34 (68)
**Liver diseases:** ***n*** **(%)**	
Alcohol-related liver cirrhosis	22 (44)
Acute liver failure^b^	6 (12)
Autoimmune liver disease^c^	4 (8)
Viral hepatitis^d^	4 (8)
Liver cirrhosis of idiopathic origin	3 (6)
HCC	3 (6)
CCC	3 (6)
Other liver diseases^e^	5 (10)
**Liver cirrhosis:** ***n*** **(%)**	36 (72)
Child A/B/C	1 (2.8)/12 (33.3)/23 (63.9)
**Precipitating events for ACLF:** ***n*** **(%)**	15 (30)
Infections	9 (60)
Gastrointestinal bleedings	6 (40)
**Life support in the MICU:** ***n*** **(%)**	
Renal replacement using dialysis: required/not required	23/27 (46/54)
Mechanical ventilation: required/not required	13/37 (26/74)
• Delta-PEEP [cmH2O]: mean ± SD (range)	−0.7 ± 2 (−4.7 to 4)
• Delta-Ppeak [cmH2O]: mean ± SD (range)	−1.2 ± 4.2 (−14 to 10)
Vasoactive drugs: required/not required	29/21 (58/42)
**MELD score [points]: mean** **±SD (range)**	
Values at admission (*n* = 50)	25.2 ± 8.6 (8–40)
Absolute values (*n* = 187)	25.8 ± 9 (7–40)
Delta-values (*n* = 137)	−0.3 ± 4 (−18 to 12)
**HARI: mean** **±SD (range)**	
Values at admission (n = 50)	0.74 ± 0.07 (0.57–0.9)
Absolute values (n = 187)	0.74 ± 0.08 (0.55–0.95)
Delta-values (n = 137)	−0.003 ± 0.057 (−0.17 to 0.16)
**Maximum PVv [cm/s]: mean** **±SD (range)**	
Values at admission (*n* = 50)	19.1 ± 14.1 (−39.8 to 45.7)
Absolute values (*n* = 187)	19.2 ± 15.7 (−43.8 to 49.2)
Delta-values (*n* = 137)	0.4 ± 7 (−39.5 to 20.3)

Presentation of the baseline demographic (age, sex) and clinical (MICU stay, mortality in the MICU, liver diseases, liver cirrhosis, precipitating events for ACLF, life support in the ICU, MELD score, HARI, maximum PVv) characteristics of the total study cohort (*n* = 50). ^a^main cause of death was ACLF (*n* = 15) which resulted in septic multiorgan failure or coagulation failure with bleeding, ^b^drug-induced liver injury, ^c^autoimmune liver disease: primary sclerosing cholangitis (*n* = 2), primary biliary cholangitis (*n* = 2), ^d^viral hepatitis: hepatitis A (*n* = 1), B (*n* = 1), C (*n* = 1), B + C + E (*n* = 1), ^e^includes liver transplantation (*n* = 1), cavernous transformation of the portal vein (*n* = 1), cholangiosepsis (*n* = 1), secondary sclerosing cholangitis (*n* = 1), and hemochromatosis (*n* = 1).

The aim of the study was to assess the potential of Doppler ultrasound as a predictor of the outcome of patients with severe liver diseases and as a novel prognostic biomarker for ACLF. The study was approved by the Ethics Committee of the University of Regensburg, Regensburg, Germany (registration number 18-920-101). All patients provided written, informed consent before the study, in accordance with the principles of the Declaration of Helsinki [revision of (18)].

**Figure 1:**
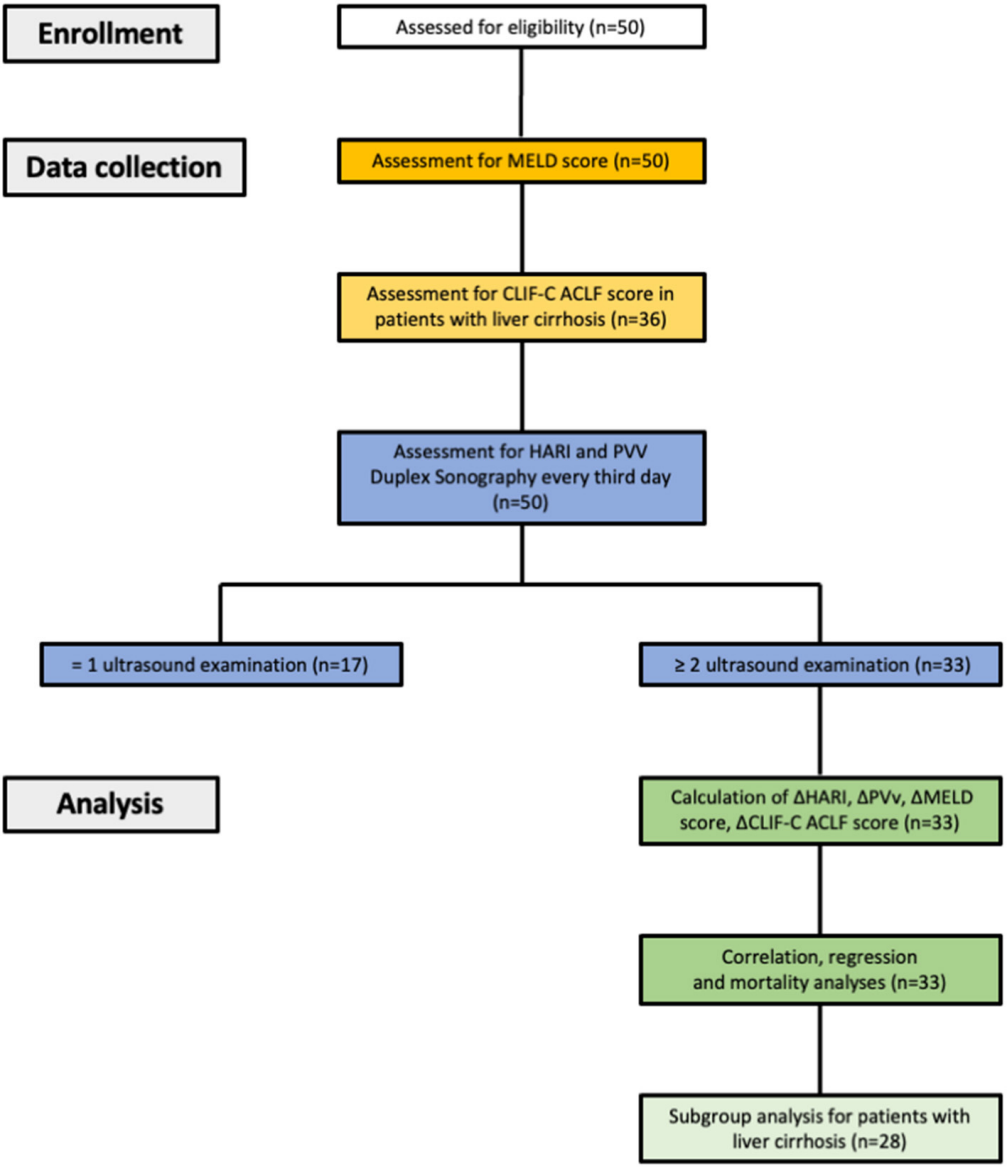
Study design, data collection and data analysis. HARI, hepatic artery resistance index; PVv, portal vein velocity; CLIF-C ACLF score, chronic liver failure-consortium acute-on-chronic liver failure score.

A flow chart of eligible patients with liver disease, data collection, and analysis is given in Figure 1.

### Ultrasound and doppler analyses and prognosis

To quantify hepatic perfusion, the hepatic artery resistance index (HARI) and the maximum portal vein velocity (PVv) were determined at admission to the MICU and then every third day using Doppler ultrasonography. A total of 187 ultrasound and Doppler examinations were performed in 50 patients (mean 3.74; range 1–12). Seventeen patients were examined once, 8 patients twice, 4 patients three times, and 21 patients four or more times. A standardized protocol was used for the positioning and breathing/ventilation of the patients during the ultrasound examination and Doppler sonography according to the literature (19, 20). We determined the hepatic artery resistance index (HARI) and maximum portal vein velocity (PVv).

**Figure 2:**
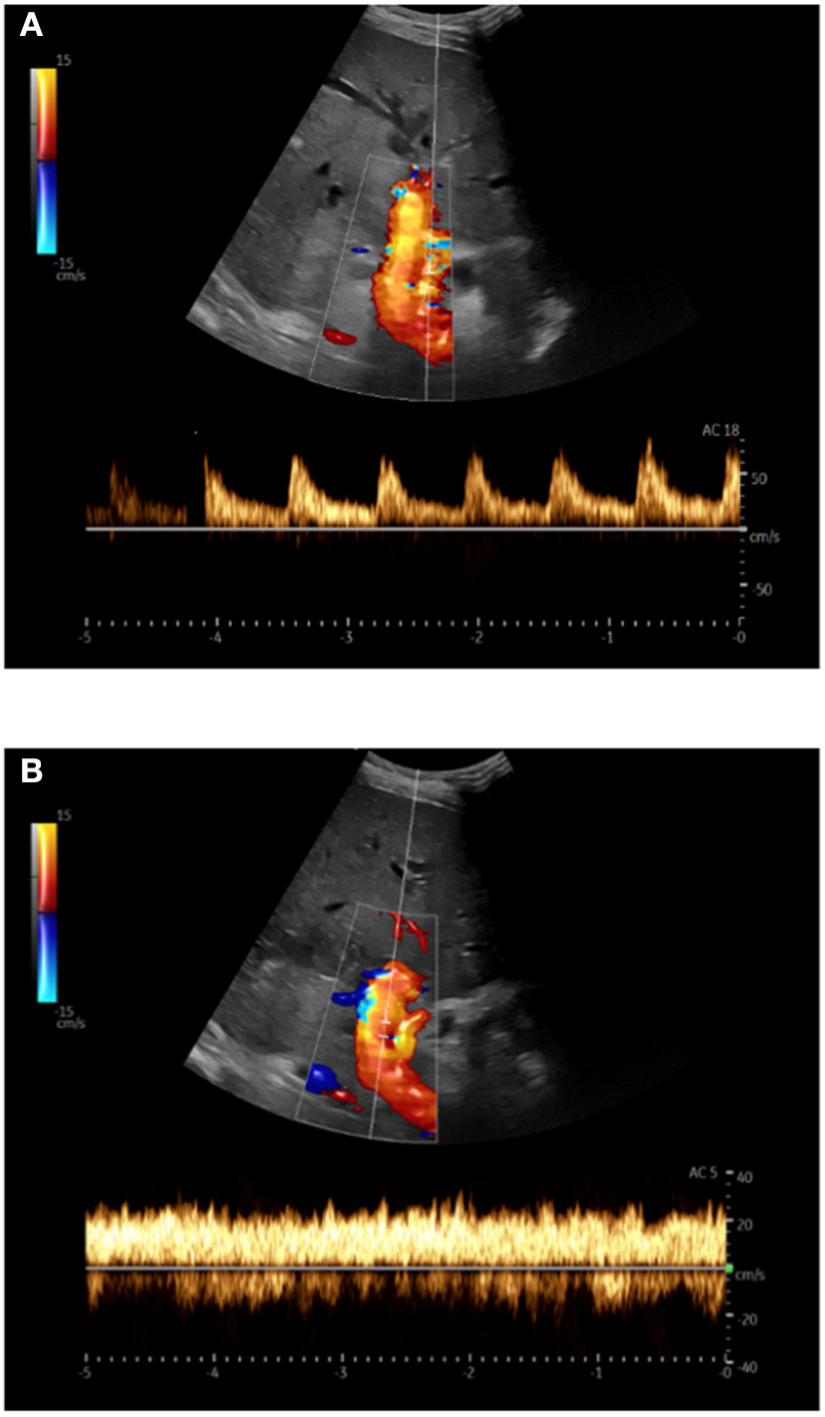
Doppler sonography in triplex technique (B-image + color Doppler + spectral Doppler) in a patient with acute-on-chronic liver failure; **(A)** Hepatic artery: Derivation of the arterial flow signal; **(B)** Portal vein: The maximum flow velocity was measured at the level of the hepatic artery after angle correction.

Ultrasound scans were performed by experienced examiners. Imaging and processing of the recordings were carried out with the mobile ultrasound system Noblus^®^ (Hitachi Aloka Medical, Ltd., Japan). The hemodynamic parameters were recorded using a convex transducer with a 1–5 MHz frequency range. Each examination consisted of three independent measurements of PVv and the HARI. The mean values were calculated and recorded. An example of a Doppler ultrasound examination in a patient with ACLF is shown in Figure 2.

The PVv was measured using Pulsed-Wave-(PW)-Doppler in the hepatoduodenal ligament at the crossing level of the proper hepatic artery and the portal vein. The HARI was recorded at the crossing level of the hepatic artery and the portal vein using PW-Doppler (Figure 2). The resistance index was automatically calculated using the following equation (21):


$$\begin{array}{l} \text{HARI}=\left(\text{Peak systolic velocity}-\text{End}-\text{diastolic velocity}\right)/\\ \text{Peak systolic velocity}. \end{array}$$

The MELD score, a well-established indicator of the mortality of patients with end-stage liver disease, was calculated for each patient—simultaneously with the ultrasound examination—using the following equation (22, 23):


$$\begin{array}{l} \text{MELD score}=9.57\times \text{ln }\left(\text{serum creatinine}\right)\\ +3.78\text{ ln }\left(\text{total bilirubin}\right)\;+11.2\\ \times \text{ln }\left(\text{international normalized ratio}\right)+\;6.43 \end{array}$$

In patients with decompensated liver cirrhosis—in addition to the MELD score—the Chronic Liver Failure Consortium (CLIF)-C ACLF score, a score derived and validated by the CLIF consortium, was determined to predict the mortality of patients with ACLF (24). The following formula was used for the calculation, wherein CLIF-C OF score was raised according to (24):


$$\begin{array}{l} \text{CLIF}-\text{C ACLF score=10}\times \;(\text{0},\text{33}\times \text{CLIF}-\text{C}-\text{OFs+0},\text{04}\\ \times \text{Age+0},\text{63}\times \text{ ln}(\text{WBC count in }10^{3}\text{/μl})-\text{2}) \end{array}$$

To analyze the impact of life support in the MICU on liver perfusion, we recorded whether invasive ventilation (Servo-i^®^, Getinge, Sweden), renal replacement therapy (multiFiltrate Ci-Ca^®^, Fresenius, United States of America) or catecholamine therapy was required during intensive care treatment. Ventilation was pressure-controlled or pressure-supported; positive end-expiratory pressure (PEEP) and peak pressure (Ppeak) at the time of the ultrasound examination were collected. None of the patients received non-invasive ventilation during the ultrasound examination. The continuously administered catecholamines during the ultrasound examination were recorded in their respective dosage (Norepinephrine in mg/h, epinephrine in mg/h, dobutamine in mg/h, terlipressin in mcg/h, and vasopressin in IU/h).

### Statistical analyses

Data were analyzed using SPSS Statistics, version 25 (IBM, USA). Correlation analyses of perfusion parameters, the MELD score, and the CLIF-C ACLF score were performed according to Pearson. The strength and direction of the correlations were described by the determined correlation coefficient (*r*). In addition, linear regression analyses of the perfusion parameters and the MELD score were carried out and described using the *R*^2^-value. As part of the study, differences in parameters were determined for specific groups. Mann-Whitney-*U*-tests were used for non-normally distributed variables and *t*-tests for normally distributed variables with equal variance. A *p*-value of 0.05 was set as the level of significance. Multiple regression analyses were performed to examine the extent to which liver perfusion was affected by life support at the MICU.

## Results

### Baseline characteristics of the patients

A total of 50 patients were enrolled in the study. The demographic and clinical characteristics of these 50 study patients are summarized in Table 1. Thirty-four patients were male, and 16 were female. The age of the cohort ranged from 39 to 90 years (mean 59.7; SD ± 10.3 years). The study included patients with different stages of acute and chronic liver diseases. Thirty-six patients were diagnosed with liver cirrhosis. The leading etiology of liver cirrhosis was alcohol-related (*n* = 25), which was diagnosed in 3 patients with hepatocellular carcinoma (HCC). Other causes of liver cirrhosis were autoimmune liver diseases (*n* = 4), viral hepatitis (*n* = 4), and cirrhosis of idiopathic origin (*n* = 3). The patients with liver cirrhosis were categorized according to the Child-Pugh classification, 1 patient was classified with liver cirrhosis Child-Pugh A, 12 patients with liver cirrhosis Child-Pugh B, and 23 patients with liver cirrhosis Child-Pugh C. In summary, the majority of our patient cohort had advanced stages of liver cirrhosis and were at high risk of developing acute-on-chronic liver failure (Table 1).

The patients without underlying liver cirrhosis (*n* = 14) had been admitted to the MICU due to drug-induced acute liver failure (ALF) (*n* = 6), cholangiosepsis (*n* = 5), and liver diseases of different etiologies (*n* = 3).

On average, the patients were treated at the MICU for 15.3 (SD ± 13.8) days. The length of the MICU stay ranged from a minimum of 2 days to a maximum of 72 days. Twenty-three of the 50 patients studied (=46%) underwent renal replacement therapy, and 13 patients (=26%) required mechanical ventilation therapy during intensive care treatment. Twenty-nine patients (=58%) required vasoactive medication for circulatory support during the MICU stay. Sixteen of the 50 examined patients (32%) died during the MICU stay. The leading cause of death was acute-on-chronic liver failure (93% of deceased patients, *n* = 15). In the non-survivors, ACLF resulted, despite maximum intensive care therapy, in multiorgan failure, coagulation failure, and circulation failure (15/16 patients). One patient (1/16 patients) newly diagnosed with congestive hepatopathy died of septic shock due to severe pneumonia.

Precipitating events for ACLF were infections and gastrointestinal bleedings. In 9 patients, infections were the precipitating events for ACLF (6/9 patients with pneumonia and 3/9 patients with urosepsis). In 6 patients, gastrointestinal bleeding was the precipitating events for ACLF (4/6 patients with varicose bleeding and 2/6 patients with non-varicose upper gastrointestinal bleeding).

HARI, PVv, and MELD score were collected and analyzed at the time of admission to the ICU. Overall *n* = 50 patients were studied. On average, the HARI was 0.74, the maximum PVv was 19.1 cm/s, and the MELD score was 25.2 at admission (Table 1). The mean values of HARI, PVv, and MELD score were compared for deceased and survived patients by *t*-test and Mann-Whitney *U*-test, respectively (Table 4A). On admission to the ICU, non-survivors had, on average, a higher MELD score (29.7 vs. 23.2, *p* = 0.010). Liver perfusion at admission did not differ significantly between non-survivors and survivors [HARI (0.75 vs. 0.73, *p* = 0.342), PVv (14.4 cm/s vs. 21.3 cm/s, *p* = 0.129)].

### Dynamic changes of the liver perfusion parameters HARI and PVv

The primary goal of our study was to analyze the changes over time of the perfusion parameters HARI and PVv and to investigate their utility as prognostic biomarkers. In patients (*n* = 33) who were examined more than once during their MICU stay, the course over time parameters (*n* = 137) was calculated from the varying absolute values of each examination and are further referred to as delta-values (Table 1). The mean of the MELD score of the patients during their MICU stay was 25.8 points and decreased by 0.3 points with each examination. The mean HARI was 0.74 and decreased by 0.003 during the MICU stay. In contrast, the mean maximum PVv was 19.2 cm/s and increased by 0.4 cm/s during the MICU stay.

### Liver perfusion parameters and the MELD score

**Figure 3:**
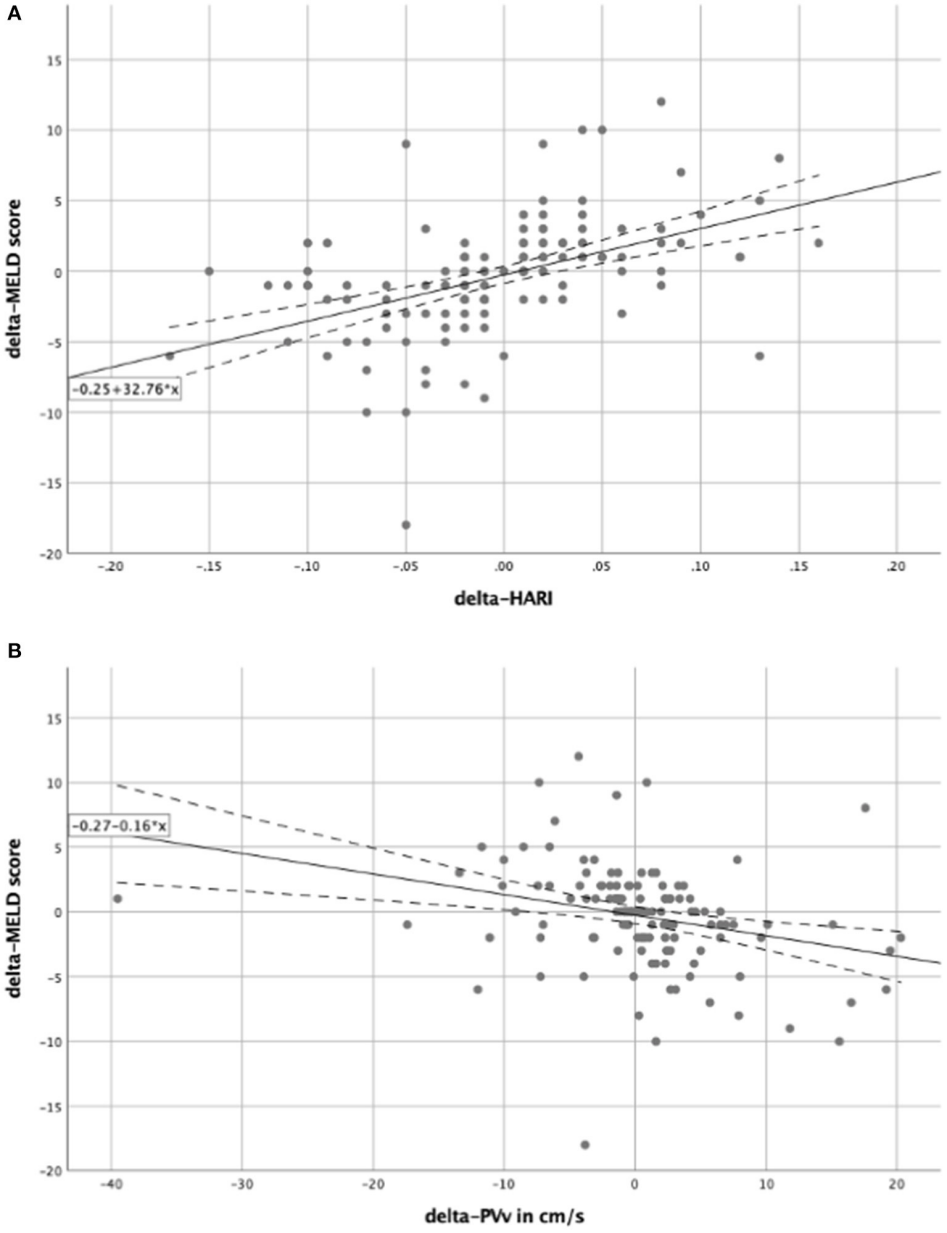
Scatter plots for the correlation between delta-MELD score and the perfusion parameters. In both scatter plots, the solid line represents the regression equation, which is also shown in the box. The dashed lines equate to the 95% confidence interval. **(A)** Showing a positive linear correlation between the delta-MELD score and the delta-HARI; **(B)** showing a negative linear correlation between the delta-MELD score and delta-PVv. HARI, hepatic artery resistance index; PVv, portal vein velocity.

The prognostic value of routine Doppler evaluation of hepatic perfusion on the MICU was determined by means of a correlation analysis of the delta-HARI and delta-PVv with the delta-MELD score. Correlation analysis was performed by bivariate correlation analyses according to Pearson (Table 2). There was a significant positive linear correlation between the delta-MELD score and the delta-HARI (*r* = 0.469; *p* < 0.001) and a negative linear correlation between the delta-MELD score and delta-PVv (*r* = −0.279, *p* = 0.001). The correlations between delta-MELD score, delta-HARI, and delta-PVv are shown in scatter plots in Figure 3. In summary, patients with increasing HARI or decreasing PVv showed an increase in their MELD Score, which reflects the worsening of their liver disease.

**Table 2:** Correlation and regression analyses between perfusion parameters and delta-MELD score.

**Analyses**	**Statistical parameter**	**Delta-HARI** **(*n* = 137)**	**Delta-PVv** **(*n* = 137)**
Correlation^a^ with the delta-MELD score	Correlation coeff. *r*	0.469	−0.279
	*P*-value	0.007 × 10^−6^*	0.001*
Regression^b^ with the delta-MELD score	*R*-value	0.469	0.279
	*R*^2^-value	0.22	0.078
	*P*-value of regression model	0.007 × 10^−6^	0.001
	Coeff. of constant	−0.25	−0.27
	Regression coeff.	32.76	−0.16
	*P*-value of regression coeff.	0.007 × 10^−6^	0.001
	95% confidence interval	22.27–43.25	−0.25 to −0.07

Results of the statistical analyses on the association between the delta-MELD score and the perfusion parameters delta-HARI and delta-PVv. ^a^Correlation analyses according to Pearson for the delta-HARI/delta-PVv and the delta-MELD score. For each correlation, the coefficient (*r*) and the *p*-value are listed. ^b^Regression analyses for the delta-HARI/delta-PVv and the delta-MELD score. For each regression, the *R*-/*R*^2^-value, the *p*-value of the regression model, the constant coefficient, the regression coefficient with its *p*-value, and the 95% confidence interval are listed. ^*^The correlations are statistically significant at the level of 0.05.

### Relation of liver perfusion parameters and the MELD score

To further investigate the influence of the delta-HARI and delta-PVv on the delta-MELD score, regression analyses (Table 2) showed an *R*^2^-value of 0.220 and 0.078. Both regression models presented *p*-values of < 0.05. The regression of the delta-HARI and the delta-MELD score resulted in a regression coefficient of 32.76 with a *p*-value of < 0.001 and a 95% confidence interval ranging from 22.27 to 43.25. The regression coefficient for delta-PVv and the delta-MELD score was −0.16 with a *p*-value of 0.001 and a 95% confidence interval ranging from −0.25 to −0.07. The determined coefficients were used to set up the following regression equations to be able to predict the course over time of the MELD score as a function of the delta-HARI and delta-PVv:

delta-MELD score = −0.25 + 32.76 × delta-HARI (Figure 3A).
delta-MELD score = −0.27−0.16 × delta-PVv (Figure 3B).

Furthermore, the relation between delta-HARI and delta-PVv was analyzed by bivariate correlation analyses according to Pearson, *r* = −0.159, *p* = 0.063. This suggests that the hepatic artery buffer response (HABR) is impaired in our patient cohort. If the HABR were functional, we would expect a positive correlation (10).

### Hepatic perfusion as a predictor of ACLF and mortality at the MICU

**Figure 4:**
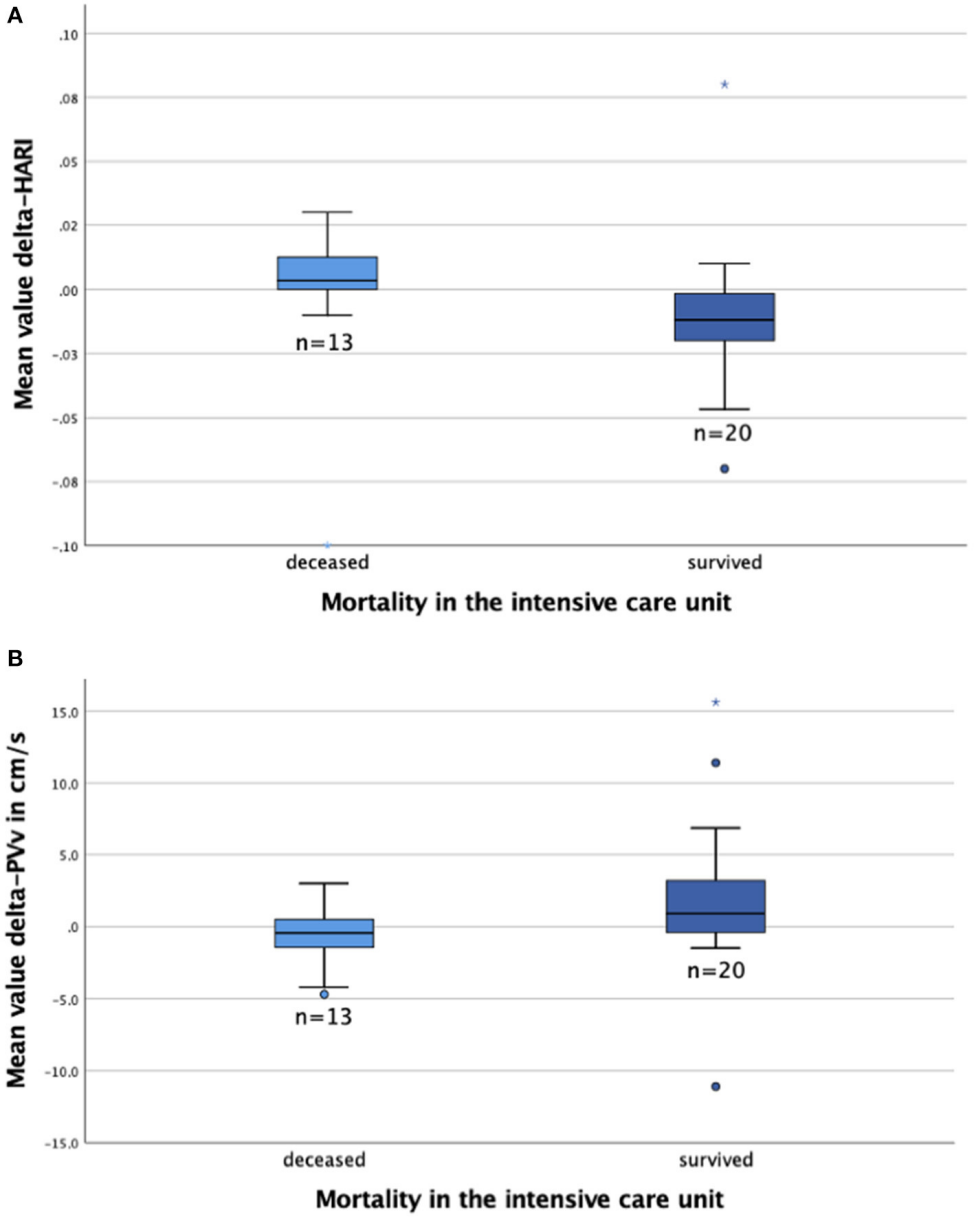
Boxplots to compare the MELD score and perfusion parameters for deceased (*n* = 13) and survived (*n* = 20) patients. The figures show the median (line in the middle of the box), 1^st^/3^rd^ quartile (lower/upper edge of the box), minimum (lower whisker), maximum (upper whisker) and outliers*°. **(A)** Delta-HARI is higher in deceased patients than in survived patients; **(B)** Delta-PVv is lower in deceased patients than survived patients. HARI, hepatic artery resistance index; PVv, portal vein velocity.

The mean values of delta-HARI, delta-PVv, and delta-MELD score were calculated for the 33 patients who had been examined more than once during their MICU stay. The mean values of these parameters were then compared to evaluate their utility as prognostic markers in patients with severe liver disease (Table 3). Patients who did not survive ACLF were characterized by an increase of their MELD score on average by 1.3 points and of the hepatic artery resistance index by 0.01, whereas maximum portal vein velocity decreased on average by 0.7 cm/s per examination. In survivors, the MELD score decreased on average by 1.9 points and the hepatic artery resistance index by 0.005, whereas maximum portal vein velocity increased on average by 1.9 cm/s per examination. The distribution of delta-HARI, delta-PVv, and delta-MELD score for non-survivors and patients who recovered is shown in boxplots in Figure 4. The comparison of non-survivors and survivors showed statistically significant differences in the mean values of delta-HARI (*p* = 0.015), delta-PVv (*p* = 0.037), and delta-MELD score (*p* = 0.002). Of clinical relevance, each of the three parameters was useful as a prognostic biomarker for patients with ACLF. Cohen's d was calculated and interpreted to quantify the size of each effect. Figure 4 shows the newly described statistically significant differences in the mean values of delta-HARI (Figure 4A) and delta-PVv (Figure 4B) between deceased and surviving patients.

**Table 3A:** Liver perfusion parameters as predictors of mortality.

**Parameters**	**Deceased patients (*n* = 16)**	**Survived patients (*n* = 34)**	***P*-value**	**Cohens's d^a^**
**(A) Liver perfusion parameters at admission as predictors of mortality**				
HARI at admission	0.75 ± 0.06 (0.65–0.84)	0.73 ± 0.08 (0.57–0.9)	0.342^†^	0.291 (weak)
PVv at admission	14.4 ± 15 (−20.9 to 29.9)	21.3 ± 13.2 (−39.8 to 45.7)	0.129*	0.44 (weak)
MELD score at admission	29.7 ± 6.2 (21–40)	23.2 ± 8.8 (8–40)	0.01^†^	0.809 (strong)

Mean comparison of HARI, PVv and MELD score at admission for deceased and survived patients. The results are expressed as mean ± SD (range). Only the MELD score at admission shows statistically significant differences in its means values. ^a^Cohen's d indicates effect size, calculated using https://www.psychometrica.de/effektstaerke.html, ^*^Mann-Whitney-*U*-test used for non-normally distributed PVv at admission,^†^*t*-test used for normally distributed HARI and MELD score at admission.

**Table 3B:** Differences in perfusion parameters over time predicting mortality.

**Parameters**	**Deceased** **patients (*n* = 13)**	**Survived** **patients (*n* = 20)**	***P*-value**	**Cohens's d^a^**
Delta-HARI	0.01 ± 0.06 (−0.1 to 0.16)	−0.005 ± 0.043 (−0.07 to 0.13)	0.015*	0.934 (strong)
Delta-PVv	−0.7 ± 2.1 (−4.7 to 3)	1.9 ± 5.3 (−11.1 to 15.6)	0.037*	0.778 (middle)
Delta-MELD score	1.3 ± 2.3 (−2 to 6)	−1.9 ± 2.9 (−10 to 3)	0.002^†^	1.203 (strong)

Mean comparison of delta-HARI, delta-PVv and delta-MELD score for deceased and survived patients. The results are expressed as mean ± SD (range). The *p*-value of each of the three parameters shows statistically significant differences in their means. ^a^Cohen's d indicates effect size, calculated *via* https://www.psychometrica.de/effektstaerke.html, ^*^Mann-Whitney-U-test used for non-normally distributed delta-HARI and delta-PVv,^†^*t*-test used for normally distributed delta-MELD score.

**Figure 5:**
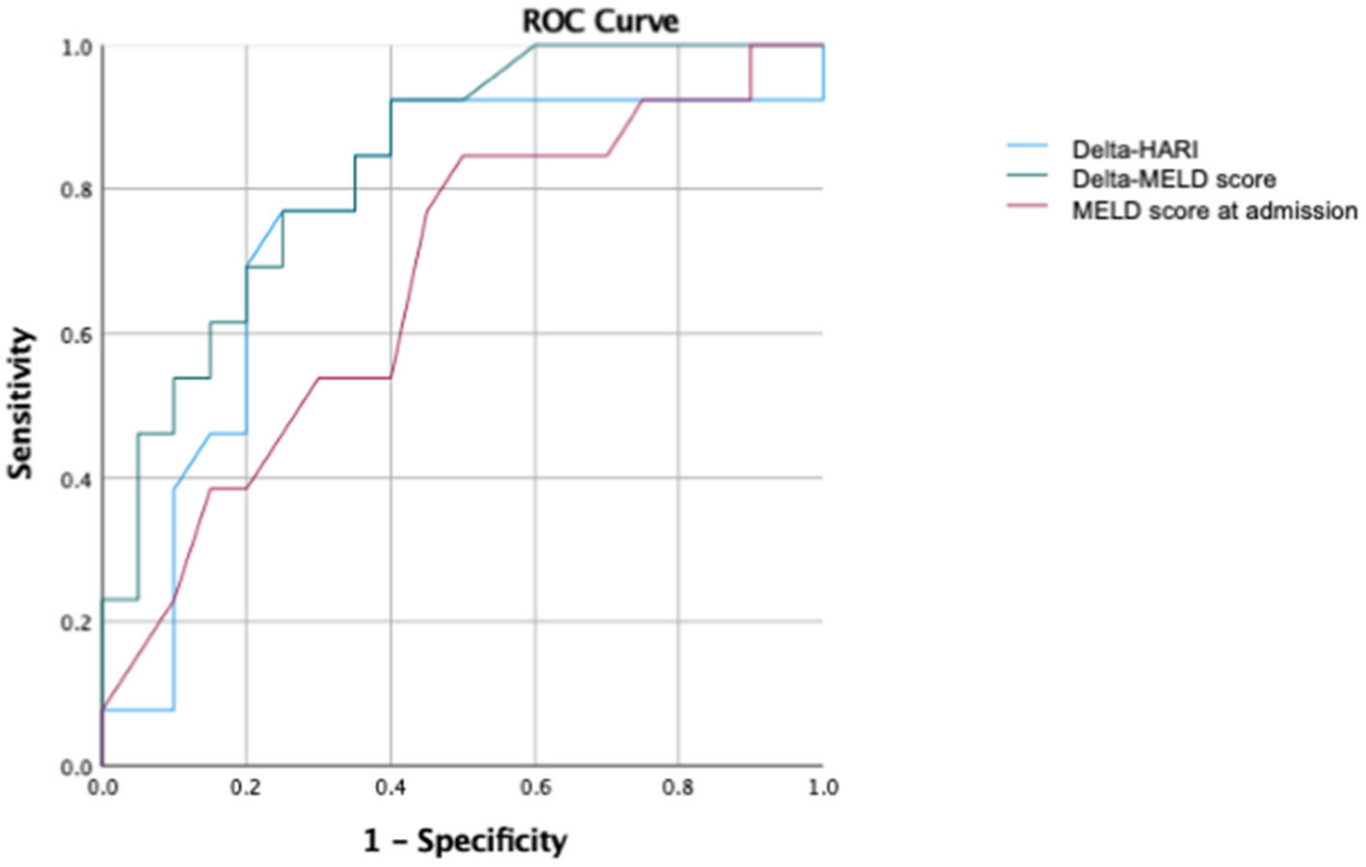
ROC analysis for the prediction of ICU mortality. AUC of delta-HARI was 0.76 (95% Confidence Interval: 0.58–0.94, *p* = 0.012), AUC of delta-MELD was 0.84 (95% Confidence Interval: 0.70–0.97, *p* = 0.01), and AUC of MELD-Score at admission was 0.71 (95% Confidence Interval: 0.53–0.89, *p* = 0,049).

Thus, delta-HARI and delta-PVv can predict the mortality of critical care patients with severe liver diseases to a similar extent as the delta-MELD score. Differences in the mean values of the delta-MELD score indicate a slightly stronger effect on mortality and higher prognostic predictive value of the delta-MELD in comparison to the delta-HARI. In our dataset, the area under the curve (AUC) for the prediction of ICU mortality for delta-HARI was 0.76 (95% Confidence Interval: 0.58–0.94, *p* = 0.012) and thus only slightly lower than that of the delta-MELD with an AUC of 0.84 (95% Confidence Interval: 0.70–0.97, *p* = 0.01). Increasing HARI and decreasing PVv are early predictors of an adverse outcome of patients with severe liver diseases. A summary of the data is given in Figure 5.

### Effect of life support in the MICU on liver perfusion

Multiple regression analyses were performed to examine the extent to which delta-HARI and delta-PVv were affected by factors other than the delta-MELD score. Here, we primarily focused on the effects of intensive care therapeutic procedures.

Forty-six percentage of the patients underwent renal replacement therapy (Table 1). In our study, no ultrasound examinations were performed while the patients were dialyzed. Liver perfusion did not significantly differ in patients with dialysis or without dialysis (delta-HARI 0.006 vs. −0.008, *p* = 0.074, delta-PVv 0.8 cm/s vs. 0.9 cm/s, *p* = 0.868, Mann-Whitney *U*-test). Twenty-six percentage of the patients required mechanical ventilation during intensive care treatment. There was no correlation between ventilation pressures (PEEP and Ppeak) and liver perfusion over time. Fifty-eight percentage of the patients required vasoactive medication for circulatory support during the ICU stay, with norepinephrine being the most frequently used catecholamine. A combination therapy of vasoactive agents was required in 11 of the 29 patients receiving vasoactive medication. Delta-norepinephrine (*r* = 0.247, *p* = 0.004) and delta-epinephrine (*r* = 0.244, *p* = 0.004) showed a positive correlation with the delta-HARI. Delta-dobutamine (*r* = −0.18, *p* = 0.036) correlated statistically significantly with delta-PVv. Details concerning the effect of life support in the MICU on liver perfusion are shown in Table 4A.

**Table 4A:** Life support and liver perfusion.

**Life support in the MICU**		**Delta-HARI**		**Delta-PVv**	
**(A) Effect of life support in the MICU on liver perfusion**					
Renal replacement therapy	Required (*n* = 23)	0.006 ± 0.06 (−0.1 to 0.16)		0.8 ± 4.1 (−4.7 to 15.6)	
	Not required (*n* = 27)	−0.008 ± 0.03 (−0.04 to 0.08)		0.9 ± 5.2 (−11.1 to 11.4)	
	*P*-value	0.074^*^		0.868^*^	
Mechanical ventilation		Delta-PEEP	Delta-Ppeak	Delta-PEEP	Delta-Ppeak
	Correlation^a^ coeff. r	0.086	0.08	0.156	0.016
	*P*-value	0.522	0.551	0.243	0.906
	Required (*n* = 13)	−0.01 ± 0.01 (−0.04 to 0.01)		−0.2 ± 4.1 (−11.1 to 6.9)	
	Not required (*n* = 37)	0.01 ± 0.06 (−0.01 to 0.16)		1.5 ± 4.8 (−4.7 to 15.6)	
	*P*-value	0.321^*^		0.75^*^	
Vasopressor therapy		Delta-Norepinephrine	Delta-Epinephrin	Delta-Dobutamine	
	Correlation^b^ coeff. r	0.247	0.244	−0.18	
	*P*-value	0.004^†^	0.004^†^	0.036^†^	

Effect of renal replacement therapy, mechanical ventilation, and vasopressor therapy on delta-HARI and delta-PVv. Comparison of the mean value of delta-HARI and delta-PVv for patients who require renal replacement therapy or mechanical ventilation and for those who do not. No statistically significant differences could be found. ^*^Mann-Whitney-U test was used for non-normally distributed delta-HARI and delta-PVv. ^a^Pearson's correlation analyses for delta-HARI/delta-PVv and delta-PEEP and delta-Ppeak, coefficient (r) and *p*-value given for each correlation. ^b^Pearson's correlation analyses for delta-HARI and delta-Norepinephrine and delta-Epinephrine as well as delta-PVv and delta-Dobutamine. Statistically significant correlations between delta-HARI and delta-PVv with vasopressors are shown.^†^Correlations are statistically significant (*p* < 0.05).

In multiple regression analyses, factors that potentially influence liver perfusion such as delta-norepinephrine, delta-epinephrine, delta-dobutamine, delta-terlipressin, delta-vasopressin, delta-PEEP, and delta-Ppeak were compared with the delta-MELD score concerning their effect on the respective liver perfusion parameters. The regression for delta-HARI yielded a corrected *R*^2^ of 0.290 with a model significance of *p* < 0.001. Delta-MELD score (*p* < 0.001), delta-norepinephrine (*p* = 0.033), and delta-dobutamine (*p* = 0.027) showed a significant effect on the course of HARI, with delta-MELD score clearly exerting the most significant influence (standardized coefficient beta: 0.439 vs. 0.189 and 0.168, respectively) (Table 4B). The regression for delta-PVv yielded a corrected *R*^2^ of 0.116 with a model significance of *p* = 0.003. For delta-MELD score (*p* = 0.001) and delta-dobutamine (*p* = 0.010) a significant effect on delta-PVv could be determined. Again, delta-MELD score affected the course of PVv more pronounced than delta-dobutamine (standardized coefficient beta: −0.281 vs. −0.219) (Table 4B).

**Table 4B:** Multiple regression analyses between life support in the MICU and liver perfusion.

**Life support**	**Delta-HARI**			**Delta-PVv**		
	**Stand. coeff**.	***P*-value**	**95% confidence interval**	**Stand. coeff**.	***P*-value**	**95% confidence interval**
Delta-PEEP	0.112	0.565	−0.021 to 0.043	0.04	0.063	−0.044 to 1.697
Delta-Ppeak	−0.285	0.155	−0.004 to 0.001	−0.024	0.28	−0.538 to 0.157
Delta-Norepinephrine	0.189	0.033^*^	0.002–0.037	0.067	0.495	−1.6 to 3.293
Delta-Epinephrine	0.153	0.211	−0.098 to 0.440	−0.06	0.659	−45.248 to 28.718
Delta-Terlipressin	0.063	0.422	−0.0001 to 0.0002	−0.085	0.338	−0.032 to 0.011
Delta-Dobutamine	0.168	0.027^*^	0.001–0.014	−0.219	0.01^*^	−2.116 to 0.29
Delta-Vasopressin	0.087	0.497	−0.021 to 0.043	−0.056	0.695	−5.334 to 3.567
Delta-MELD score	0.439	0.007 × 10^−6*^	0.004–0.008	−0.281	0.001^*^	−0.797 to 0.196

Results of the multiple regression analyses between perfusion parameters delta-HARI and delta-PVv and potentially influencing factors of the life support in the MICU. For each regression, the standardized coefficient beta, the *p*-value, and the 95% confidence interval are given. Delta-MELD score shows the most significant and highest effect on delta-HARI and delta-PVv. ^*^The regressions are statistically significant (*p* < 0.05).

In summary, delta-HARI and delta-PVv are significantly influenced by the delta-MELD score and not by dialysis or mechanical ventilation. Norepinephrine and dobutamine have a mild to moderate impact on liver perfusion, while the delta-MELD score exerts the most significant effect on the course of the perfusion parameters HARI and PVv. Our analyses also reveal that optimized catecholamine therapy and fluid management are potential therapeutic targets to improve liver perfusion.

### Analysis of liver perfusion in patients with liver cirrhosis—Comparison with CLIF-C ACLF and MELD score and early prediction of mortality in the MICU

Of clinical relevance, the leading cause of death in our patient cohort was an acute-on-chronic liver failure (93% of deceased patients, *n* = 15). Therefore, we performed a correlation analysis between delta-HARI and delta-PVv with the delta-MELD and delta-CLIF-C ACLF score in a subgroup analysis of the 36 patients with liver cirrhosis.

The CLIF-C ACLF score is a score that was derived and validated by the chronic liver failure (CLIF) consortium to predict the mortality of patients with ACLF (24). The CLIF-C ACLF score combines the age of the patient and the white blood cell count with the chronic liver failure (CLIF) organ failure score (CLIF OF score), which is a modified version of the Sequential Organ Failure Assessment (SOFA) score (24–26). The CLIF OF score system comprises the organs/systems liver, kidney, brain, coagulation, circulatory, and respiratory with the respective subscores 1–3 (24).

For the subgroup analysis of patients with liver cirrhosis (*n* = 33), HARI, PVv, and MELD score were collected at admission to the MICU and compared for deceased and survived patients by *t*-test and Mann-Whitney *U*-test, respectively (Table 6A). Analogous to the complete patient collective, the liver perfusion parameters at admission showed no significant differences regarding the mortality of the patients. MELD score was a predictor of mortality on admission.

### Dynamic changes over time of the liver perfusion parameters in patients with liver cirrhosis

Changes over time in the liver perfusion parameters were analyzed in the subgroup of patients with liver cirrhosis. Our analyses showed a significant positive linear correlation between the delta-HARI and the delta-MELD score (*r* = 0.517; *p* < 0.001) and the delta-CLIF-C ACLF score (*r* = 0.252; *p* = 0.005). In addition, we could demonstrate a concomitant negative linear correlation between delta-PVv and the delta-MELD score (*r* = −0.316; *p* < 0.001).

There was no significant correlation between delta-PVv and delta-CLIF-C ACLF score (*r* = −0.106, *p* = 0.246).

For further investigation of the correlation of delta-HARI or delta-PVv with the delta-MELD score and the delta-CLIF-C ACLF score, regression analyses were performed. These showed an *R*^2^-value of 0.261 for the influence of delta-HARI on the delta-MELD score and an *R*^2^-value of 0.063 for the influence of delta-HARI on delta-CLIF-C ACLF score, respectively. Both regression models showed *p*-values of < 0.05. For the effect of the delta-PVv on the delta-MELD score an *R*^2^-value of 0.1 was calculated (*p* < 0.05).

Regression analyses for the effect of delta-PVv on the delta-CLIF-C ACLF score showed *R*^2^-values of 0.011 and were not significant, *p* = 0.245.

**Figure 6:**
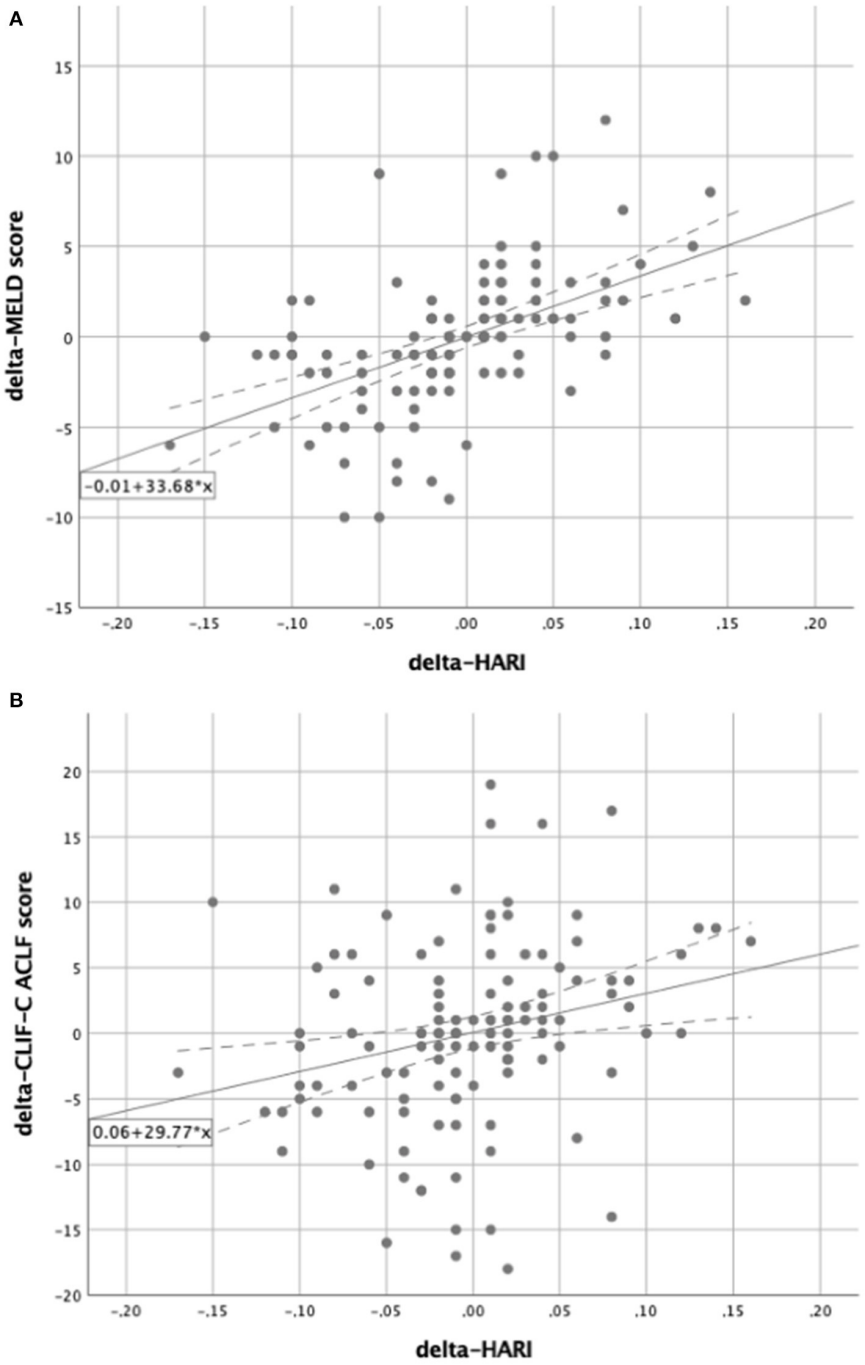
Subgroup analysis for patients with liver cirrhosis. Scatter plots for the correlation between delta-HARI and delta-MELD or delta-CLIF-C ACLF score for patients with liver cirrhosis. **(A)** Showing a positive linear correlation between delta-HARI and delta-MELD score. **(B)** showing a positive linear correlation between delta-HARI and delta-CLIF-C ACLF score.

Data are shown in Tables 5, 6 and Figure 6.

**Table 5:** Subgroup analyses—Correlation and regression analyses between perfusion parameters and delta-MELD score and delta-CLIF-C ACLF score for patients with liver cirrhosis.

**Analyses**	**Statistical parameter**	**Delta-HARI** **(*n* = 122)**	**Delta-PVv** **(*n* = 122)**
Correlation^a^ with the delta-MELD score	Correlation coeff. R	0.517	−0.316
	*P*-value	1.070 × 10^−9^*	3.870 × 10^−4^*
Regression^b^ with the delta-MELD score	*R*-value	0.517	0.316
	*R*^2^-value	0.267	0.1
	*P*-value of regression model	1.070 × 10^−9^	3.870 × 10^−4^
	Coeff. of constant	−0.01	−0.07
	Regression coeff.	33.68	−0.17
	*P*-value of regression coeff.	1.070 × 10^−9^	3.870 × 10^−4^
	95% confidence interval	23.60–43.75	−0.25 to −0.08
Correlation^a^ with the delta-CLIF-C ACLF score	Correlation coeff. r	0.252	−0.106
	*P*-value	0.005*	0.245
Regression^b^ with the delta-CLIF-C ACLF score	*R*-value	0.252	0.106
	*R*^2^-value	0.063	0.011
	*p*-value of regression model	0.005	0.245
	Coeff. of constant	0.06	−4 × 10^−3^
	Regression coeff.	29.77	−0.1
	*P*-value of regression coeff.	0.005	0.245
	95% confidence interval	9.1–50.45	−0.27 to −0.07

Results of the correlation and regression analyses between perfusion parameters delta-HARI and delta-PVv and delta-MELD score and delta-CLIF-C ACLF for patients with liver cirrhosis. ^a^Correlation analyses according to Pearson for delta-HARI/delta-PVv and delta-MELD score and delta-CLIF-C ACLF score. For each correlation, the coefficient (*r*) and the *p*-value are listed. ^b^Regression analyses for the delta-HARI/delta-PVv and the delta-MELD score and the delta-CLIF-C ACLF score, respectively. For each regression, the *R*-/R^2^-value, the *p*-value of the regression model, the constant coefficient, the regression coefficient with its *p*-value, and the 95% confidence interval are listed. ^*^The correlations are statistically significant at the level of 0.05.

**Table 6A:** Subgroup analyses—Liver perfusion parameters as predictors of mortality in patients with liver cirrhosis.

**Parameters**	**Deceased patients** **(*n* = 15)**	**Survived patients** **(*n* = 21)**	***P*-value**	**Cohens's d^a^**
**(A) Subgroup analyses—Perfusion parameters at admission as predictors of mortality in patients with liver cirrhosis**				
HARI at admission	0.75 ± 0.06 (0.65–0.84)	0.75 ± 0.06 (0.61–0.85)	0.952^†^	0.021 (no effect)
PVv at admission	14.3 ± 15.6 (−20.9 to 29.9)	22.4 ± 8.2 (8.5–45.7)	0.446*	0.259 (weak)
MELD score at admission	30.0 ± 6.3 (21–40)	24.0 ± 8.3 (9–40)	0.023^†^	0.803 (strong)

Mean comparison of HARI, PVv and MELD score at admission for deceased and survived patients with liver cirrhosis. The results are given as mean ± SD (range). Only the MELD score at admission shows statistically significant differences in its means. ^a^Cohen's d indicates effect size, calculated using https://www.psychometrica.de/effektstaerke.html,^†^*t*-test used for normally distributed HARI and MELD score at admission, ^*^Mann-Whitney-*U*-test used for non-normally distributed PVv at admission.

**Table 6B:** Subgroup analyses—Differences in perfusion parameters over time predicting mortality in patients with liver cirrhosis.

**Parameters**	**Deceased patients** **(*n* = 13)**	**Survived patients** **(*n* = 15)**	***P*-value**	**Cohens's d^a^**
Delta-HARI	0.01 ± 0.05 (−0.10 to 0.16)	−0.01 ± 0.03 (−0.07 to 0.08)	0.011*	1.065 (strong)
Delta-PVv	−0.7 ± 2.1 (−4.7 to 3)	1.9 ± 6.1 (−11.1 to 15.6)	0.13*	0.6 (middle)
Delta-MELD score	1.3 ± 2.3 (−2 to 6.0)	−1.5 ± 2.8 (−10.0 to 3)	0.004*	1.239 (strong)

Mean comparison of delta-HARI, delta-PVv and delta-MELD score for deceased and survived patients with liver cirrhosis. The results are given as mean ± SD (range). The *p*-values of delta-HARI and delta-MELD score show statistically significant differences in their means. Only delta-PVv shows no significant differences in its means. ^a^Cohen's d indicates effect size, calculated *via* https://www.psychometrica.de/effektstaerke.html, ^*^Mann-Whitney-*U*-test used for non-normally distributed delta-HARI, delta-PVV and delta-MELD score.

Using the determined coefficients, the following regression equitation can predict the course over time of the delta-MELD score and the delta-CLIF-C-ACLF score as a function of delta-delta-HARI and delta-PVv.

Delta-MELD score = −0.01 + 33.68 x delta-HARI (Figure 6A).

Delta-CLIF-C ACLF score = 0.06 + 29.77 x delta-HARI (Figure 6B).

Delta-MELD score = −0.07 to 0.17 × delta-PVv

Delta-CLIF-C ACLF score was not calculated as a function of delta-PVv as the regression analyses were not significant.

In summary, in our subgroup analysis for patients with liver cirrhosis and ACLF, we could confirm the correlation of the delta-HARI and delta-PVv with the delta-MELD score, which were evident in the entire cohort. The correlations in the subgroup of patients with liver cirrhosis and ACLF were even higher than in the entire cohort, highlighting the relevance of our findings for patients with cirrhosis and ACLF.

Furthermore, we identified a new significant correlation of the delta-HARI with the delta-CLIF-C ACLF score in this cohort, which is of high clinical relevance because the CLIF-C ACLF score is -up to date- the prognostic score, which is the best predictor of mortality in ACLF (26). The correlations between delta-HARI and delta-CLIF-C ACLF are lower than the correlations between the delta-HARI and the delta-MELD score. We attribute this to the fact that all organ systems are included in the prediction of mortality of the CLIF-C ACLF score, whereas in our study, the ultrasound examinations were focused on the liver. In this context, the MELD score is more specific for the system “liver” in terms of the parameters included, which explains the better correlation of the delta-HARI with the delta-MELD score.

**Figure 7:**
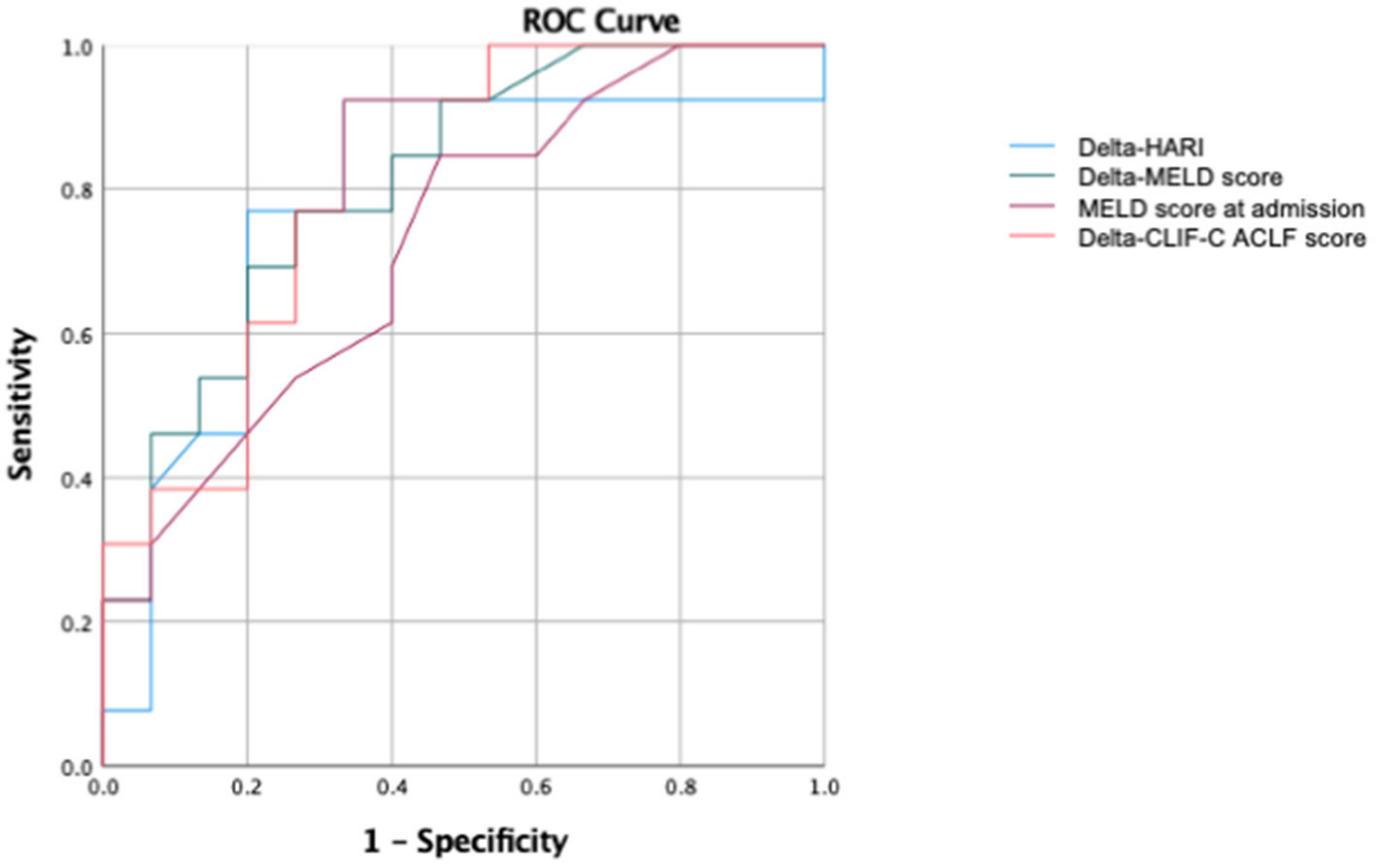
Subgroup ROC analysis for patients with liver cirrhosis of the prediction of ICU mortality. AUC of delta-HARI was 0.78 (95% Confidence Interval: 0.60–0.97, *p* = 0.011), AUC of delta-MELD was 0.81 (95% Confidence Interval: 0.65–0.97, *p* = 0.005), AUC of MELD-Score at admission was 0.73 (95% Confidence Interval: 0.54–0.914, *p* = 0.040), and AUC of delta-CLIF-C-ACLF was 0.815 (95% Confidence Interval: 0.66–0.97, *p* = 0.005).

In addition, in the subgroup of patients with liver cirrhosis, ROC (Receiver operating characteristic) analyses were performed to predict ICU mortality (Figure 7). In this subgroup of patients with liver cirrhosis, the AUC for the prediction of ICU mortality for delta-HARI was 0.78 (95% Confidence Interval: 0.60–0.97, *p* = 0.011) and thus only slightly lower than that of the delta-MELD 0.81 (95% Confidence Interval: 0.65–0.97, *p* = 0.005). The AUC of delta-CLIF-C ACLF in this subgroup was highest with 0.815 (95% Confidence Interval: 0.66–0.97, *p* = 0.005), which is in accordance with the literature regarding the CLIF-C ACLF score as the best prognostic score of ACLF.

In summary, we show a positive correlation of the delta-HARI with the delta-MELD score (*r* = 0.469, *p* < 0.001) and a negative correlation of the delta-PVv with the delta-MELD score (*r* = −0.279, *p* = 0.001). Compared with the mean values of the delta-HARI (−0.003) and the delta-PVv (0.4 cm/s), the MELD score decreased throughout the MICU stay with simultaneously decreasing resistance indices of the hepatic artery and increasing maximum portal flow velocity. As a decreasing MELD score is a positive prognostic factor, both decreasing HARI and increasing PVv can be considered as novel positive prognostic factors for patients with severe liver diseases. In contrast, increasing HARI and decreasing PVv constitute negative prognostic biomarkers for patients with severe liver diseases. These findings were confirmed in a subgroup analysis of patients with liver cirrhosis. Here we established a new correlation between the delta-HARI and the delta-CLIF-C-ACLF score (*r* = 0.252; *p* = 0.005) and confirmed the delta-HARI as an accurate predictor of the outcome of patients with ACLF.

## Discussion

This prospective cohort study aims 1. to determine the predictive value of changes in hepatic perfusion for the outcome in patients with severe liver diseases, 2. to analyze the role of liver perfusion as a new predictor for mortality due to ACLF, and 3. to establish perfusion-based biomarkers as early readouts for therapy guidance in patients with severe liver diseases and ACLF.

We have analyzed changes in hepatic perfusion of critical care patients with acute and chronic liver diseases over the course of their MICU stay to establish new biomarkers of prognosis and therapy guidance. This is the first report of a prospective time series measurement using Doppler sonography in patients with liver disease in the MICU. An increase in the hepatic artery resistance index (HARI) and a decrease in portal vein velocity (PVv) during the MICU stay predicted an adverse outcome and increased mortality.

Previous studies have focused on hepatic hemodynamics in general and on the importance of ultrasound examination in particular (27–30), and data were collected retrospectively or as part of a cross-sectional study (31–34). Only a few studies have examined the correlation between Doppler sonographic measurements of hepatic perfusion and the MELD score so far (31–33, 35, 36).

The patients included in our study showed a mean hepatic artery resistance index of 0.74 ± 0.08 (range 0.55–0.95) and a maximum portal vein velocity of 19.2 ± 15.7 cm/s (range −43.8 to 49.2 cm/s). These results are comparable to those of other studies in patients with liver cirrhosis of alcoholic vs. viral etiology (14, 21, 37, 38). In our study, the absolute mean values of the HARI and PVv reflect the momentary/current status of hepatic perfusion, whereas the time series measurement of hepatic perfusion—reflected by the parameter delta-HARI and delta-PVv—accurately describe the development of hepatic perfusion over time during hospitalization at the MICU. Our study recorded a mean of −0.003 ± 0.057 (range −0.170 to 0.160) for delta-HARI and a mean of 0.4 ± 7.0 cm/s (range −39.5 to 20.3) for delta-PVv. In patients with a good prognosis arterial resistance in the liver decreased, and the maximum portal flow velocity increased over time and with recovery. On the contrary, increasing HARI and decreasing PVv were predictors of an adverse outcome in critically ill patients with different stages of acute and chronic liver diseases. Non-survivors showed a higher delta-HARI (0.010 vs. −0.005; *p* = 0.015) and lower delta-PVv (−0.7 vs. 1.9 cm/s; *p* = 0.037) in comparison to patients who survived. Of note, it is the change over time of these perfusion parameters, which most accurately predicts outcome and mortality. Thus, we identified delta-HARI and delta-PVv as early predictors of mortality in acute and chronic liver diseases.

Mortality in our patient cohort was predominantly due to acute-on-chronic liver failure (ACLF). Worldwide, ACLF is emerging as a major cause of mortality in patients with cirrhosis and chronic liver diseases (5). A systematic review of the global burden of ACLF recently reported a prevalence among patients admitted with decompensated cirrhosis of 35% and 90-day mortality of 55% (1). Of note, the exact definition of ACLF varies worldwide (3, 39–42). In Europe, ACLF is generally defined according to the European Association for the Study of the Liver-Chronic Liver Failure (EASL-CLIF) Consortium as an acute deterioration of pre-existing chronic liver disease associated with organ failure and high short-term mortality (i.e., death < 28 days after hospital admission) (25). In collaboration with the ESAL-CLIF Consortium, Jalan et al. (24) established a prognostic score to predict mortality in patients with acute-on-chronic liver failure, the CLIF-Consortium ACLF (CLIF-C ACLF) score. This score combines the CLIF-C Organ Failure Score [a modification of the Sequential Organ Failure Assessment (SOFA) score] with two other independent predictors of mortality (age and white cell count). Compared to other prognostic scores, such as the MELD score and Child-Pugh score, the CLIF-C ACLF score is the best available score for the prediction of 28-day mortality among patients with ACLF (43, 44). Of note, none of these scores includes liver perfusion parameters.

By demonstrating an association between delta-HARI and delta-PVv with ACLF-related mortality, our study shows for the first time that the course over time of hepatic perfusion plays a crucial role in the prognosis of patients with ACLF. We were able to show a clear utility for liver hemodynamic parameters as prognostic biomarkers by comparing serial measurements of HARI with the established prognostic scores delta-CLIF-C-ACLF in the early prediction of ACLF-related mortality.

Our results are in accordance with Mehta et al. who showed that the development of ACLF and its associated inflammatory response markedly changes intrahepatic hemodynamics with a subsequent decrease in hepatic blood flow and an increase in intrahepatic resistance, which predicted mortality (45). Solís-Muñoz et al. reported that the portal vein velocity was significantly lower in acutely decompensated patients with cirrhosis who developed ACLF than in those who did not develop ACLF (46). Furthermore, our data are in line with the results of Kuroda et al. (34) who analyzed hepatic perfusion using contrast-enhanced ultrasound (CEUS) in patients with acute liver failure (ALF) and investigated its utility as a prognostic tool (47). The authors recorded the time interval (TI) between the time to peak of the hepatic artery (HA) and liver parenchyma (LP) by performing CEUS at baseline and after 7 days. TI (HA, LP) was significantly shorter in non-survivors than in survivors and emerged as the only independent factor for predicting adverse prognosis in patients with ALF, with a 94.4% sensitivity and 90.6% specificity. This underlines the importance of preventing increasing HARI and decreasing PVv and implementing serial Doppler sonographic or CEUS-bases liver perfusion measurements in managing patients with acute and chronic liver disease. The transferability to daily clinical practice and the cost-effectiveness in the guidance of treatment is undoubtedly easier using routine Doppler sonography in comparison with CEUS.

Thus, the newly established correlation between hepatic perfusion and mortality, delta-HARI and delta-PVv, may present new valuable targets in the guidance of critical care therapy for patients with severe liver diseases by optimizing hepatic perfusion, for example, through calculated volume therapy or modulation of vasoactive medication. Thus, initial fluid resuscitation in ACLF following the recommendations of the International Guidelines for the Management of Sepsis (48) could be guided by repeated Doppler sonographic measurements to restore hemodynamics and to optimize liver perfusion. The choice of resuscitation fluid in patients with cirrhosis and ACLF is unclear. This issue was addressed by Maiwall et al. who compared the efficacy and safety of 20% Albumin to plasmalyte in the treatment of sepsis-induced hypotension (49). In critically ill patients with cirrhosis and sepsis-induced hypotension 20% albumin restores arterial pressure more quickly but causes more pulmonary complications than plasmalyte. Plasmalyte is safer and can be considered for volume resuscitation in these patients. The optimal management of the critically ill patient with sepsis and cirrhosis has not been well-defined and follows guidelines made for management of patients without cirrhosis with sepsis. Despite the lack of strong evidence, we usually follow an analogous (to patients without cirrhosis) approach to sepsis management in patients with cirrhosis, including the choice of fluids, vasopressors, and antibiotics. According to our data, we suggest monitoring fluids and vasopressors using routine Doppler sonography of the liver in patients with liver cirrhosis and ACLF. Monitoring of vasopressors is central because vasoactive medication can affect liver perfusion. We performed multiple regression analyses to identify potential effectors on liver perfusion. Renal replacement therapy and mechanical ventilation did not affect HARI and PVv. The latter has been described by Saner et al. who reported no significant differences in liver transplanted patients for maximal PVv and HARI when ventilated with different PEEP settings (50). The identified correlations between liver perfusion parameters and vasoactive medication are in accordance with previous publications (51–53). Consequently, monitoring vasopressors by Doppler sonography may help prevent adverse effects on (delta) HARI and PVv.

### Limitations

This study has several limitations. First, the sample size is small. Larger-scale prospective clinical studies are needed to confirm these findings. Second, Doppler sonography is an operator-dependent method. Third, this study is a single-center observational study which yielded clinically relevant results with respect to the use of liver perfusion parameters to guide volume and catecholamine therapy in patients with severe liver disease. The limitation lies in the observational nature of the study. A follow up interventional study should be designed including multiple participating sites to validate the efficacy of Doppler sonographic measurements of liver perfusion to guide volume resuscitation and vasopressor therapy of patients with ACLF.

## Conclusions

Here, we show that delta-HARI and delta-PVv are new predictors of outcome in patients with ACLF. Furthermore, we could show that the course over time of the HARI correlates with the CLIF-C ACLF score during ICU treatment, underlining that serial measurement of liver perfusion parameter is an early predictor of mortality due to ACLF.

Our study establishes a clear utility of routine Doppler sonography evaluating hepatic perfusion in critical care patients with severe liver diseases. In addition, the correlation between hepatic perfusion and mortality, described here for the first time, may be seen as an opportunity to improve and guide the treatment of critical care patients with severe liver diseases by optimizing hepatic perfusion, for example, through balanced volume therapy or additional vasoactive medication.

We recommend introducing a regular routine Doppler sonographic evaluation of liver perfusion in critical care patients with severe liver diseases and liver cirrhosis in everyday clinical practice to assess prognosis and to guide therapeutic management.

## Data availability statement

The raw data supporting the conclusions of this article will be made available by the authors, without undue reservation.

## Ethics statement

The studies involving human participants were reviewed and approved by Ethics Committee of the University of Regensburg, Regensburg, Germany. The patients/participants provided their written informed consent to participate in this study.

## Author contributions

SSchm, JV, CM-S, SM, KG, and MM: study concept, design, drafting of the manuscript, analyses, and interpretation of data. JV, CM-S, and SSchm: acquisition of data. JV, CM-S, SM, AM, SSchl, HT, KG, MM, and SSchm: writing and critical revision of the manuscript for important intellectual content. MM: supervision. All authors contributed to the article and approved the submitted version.

## Conflict of interest

The authors declare that the research was conducted in the absence of any commercial or financial relationships that could be construed as a potential conflict of interest.

## Publisher's note

All claims expressed in this article are solely those of the authors and do not necessarily represent those of their affiliated organizations, or those of the publisher, the editors and the reviewers. Any product that may be evaluated in this article, or claim that may be made by its manufacturer, is not guaranteed or endorsed by the publisher.

## Abbreviations

ACLF: acute-on-chronic liver failure
ALF: acute liver failure
ALI: acute liver injury
CCC: cholangiocellular carcinoma
CEUS: contrast-enhanced ultrasound
CLI: chronic liver injury
CLIF-C: chronic liver failure-consortium
CT: computed tomography
EASL: European association for the study of the liver
EDV: end-diastolic velocity
HA: hepatic artery
HARI: hepatic artery resistance index
HABR: hepatic artery buffer response
HCC: hepatocellular carcinoma
LP: liver parenchyma
MELD: model for end-stage liver disease
MICU: medical intensive care unit
PEEP: positive end-expiratory pressure
Ppeak: peak pressure
PSV: peak systolic velocity
PVv: portal vein velocity
PW, doppler: pulsed-wave-doppler
ROC: receiver operating characteristic
SD: standard deviation
TI: time interval.
